# Is Cancer Diagnosis Following Emergency Admission Due to Patient Delay or Systems Failure: New Insights from a Comprehensive Primary Care Dataset

**DOI:** 10.23889/ijpds.v1i1.350

**Published:** 2017-04-19

**Authors:** Michael Yule, Rosalind Adam, Peter Murchie

**Affiliations:** 1 University of Aberdeen Medical School; 2 Centre of Academic Primary Care, University of Aberdeen

## Objectives

A significant proportion (13.9-21.8%) of cancers are diagnosed during emergency admissions to hospital. Patients who are diagnosed in this way have poorer outcomes. Cancer type, increasing age, and deprivation have shown to be associated with cancer diagnosis as an emergency. The role of primary care consulting behaviour prior to emergency presentation with a new cancer diagnosis is unclear and therefore the principle aim of the present study was to investigate this.

## Approach

All GP practices in the North East of Scotland were invited to participate. 2015 patients with a diagnosis of either colorectal, upper GI, prostate, lung, breast and melanoma were randomly selected from participating practices. A dataset was created that contained routinely collected data from primary care records from these patients. This information was consolidated with information from the NHS Grampian Cancer Care pathway database. Those diagnosed by screening were removed, leaving 1803 patients. The main characteristic examined was whether or not the patient had seen a GP with relevant symptoms in the 2 years before the consultation that resulted in their diagnosis or referral. Chi squared analysis and univariate linear regression analysis was performed on each characteristic to examine the association of independent variables with each outcome. Multivariate linear regression was then carried out across the characteristics. 

## Results

362 (20.1%) of new cancer diagnosis were by emergency. Only 16.2% (n=260) of patients who saw their GP prior to their admission were diagnosed by emergency. 54.5% (n=97) of patients who had not seen their GP prior to diagnosis were emergency diagnoses. 20.7% (n=75) of patients diagnosed by emergency admission had been referred by their GP at the time of diagnosis and were awaiting their appointment with secondary care. Patients who had been seen in primary care before their cancer diagnosis were significantly less likely to have their cancer diagnosed by emergency admission (p<0.0001).

## Conclusion

Patients who present to their GP with symptoms of cancer are much less likely to be diagnosed as a result of an emergency admission. It is therefore postulated that GPs are adequately referring patients with cancer for further investigation and diagnosis. However around a fifth of patients overall are still being diagnosed through emergency admission to hospital, and in the majority of cases this is because they are not presenting themselves to primary care. In order to rectify this we suggest that greater awareness about cancer symptoms amongst the public is necessary.

